# Imperatorin alleviated NLR family pyrin domain-containing 3 inflammasome cascade-induced synovial fibrosis and synovitis in rats with knee osteoarthritis

**DOI:** 10.1080/21655979.2021.2012949

**Published:** 2021-12-11

**Authors:** Haosheng Zhang, Liang Ding, Xiaoqing Shi, Wei Mei, Zhengquan Huang, Li Zhang, Xiaochen Li, Bo Xu, Li Zhang, Peimin Wang

**Affiliations:** a Key Laboratory for Metabolic Diseases in Chinese Medicine, First College of Clinical Medicine, Nanjing University of Chinese Medicine, Nanjing Jiangsu Province, China; b Department of Orthopedics, Zhenjiang Hospital Affiliated to Nanjing University of Chinese Medicine, Zhenjiang Jiangsu Province, China; c Department of Orthopedics, Affiliated Hospital of Nanjing University of Chinese Medicine, Nanjing Jiangsu Province, China; d Department of Orthopedics, Jiangsu Province Hospital of Chinese Medicine, Nanjing Jiangsu Province, China

**Keywords:** Imperatorin, knee osteoarthritis, synovial fibrosis, hypoxia, synovitis, NLR family pyrin domain-containing 3 inflammasome

## Abstract

We aimed to clarify the therapeutic effects of imperatorin (IMP) on knee osteoarthritis (KOA). Thirty 3-month-old SD male rats were randomly divided into Normal, monosodium iodoacetate (MIA) and MIA+IMP groups. Their synovial tissues were subjected to histopathological analysis. Primary synovial fibroblasts obtained from additional normal rats were treated by lipopolysaccharide (LPS) and then IMP. The mRNA and protein expressions of factors related to synovitis and synovial fibrosis were detected by qRT-PCR and Western blotting, respectively. The concentrations of inflammatory factors IL-1β and IL-18 were measured by ELISA. IMP reduced HIF-1α, NLR family pyrin domain-containing 3 inflammasome expression and IL-1β, IL-18 production in synovial fibroblasts induced by LPS. IMP also downregulated synovial fibrosis markers. *In vitro* study revealed that MIA-induced synovitis and synovial fibrosis were relieved by IMP. IMP relieves the inflammation associated with synovitis and synovial fibrosis. It reduces the production of pro-inflammatory mediators and cytokines and inhibits TGF-β1, TIMP-1 and VEGF expressions that promote synovial fibrosis.

## Background

Knee osteoarthritis (KOA) is a common and disabling condition that represents a substantial and increasing health burden with notable implications for the affected individuals, health-care systems, and wider socioeconomic costs [1,2]. Synovial fibrosis is an important pathological process characterized by abnormal deposition of extracellular matrix (ECM), as well as cell migration and proliferation in the occurrence and development of KOA. Synovial fibrosis is very common in the case of KOA, as the main cause for pain and joint stiffness. Upregulation of fibrogenic factors, such as TGF-β1 [3], are signs of the development of synovial fibrosis. TGF-β1 is the most well-known fibrosis factor, and plays a key role in many profibrotic processes, including promoting tissue matrix metalloproteinase (TIMP) expressions [4]. TIMPs are elevated in some diseases related to fibrosis (e.g. liver fibrosis), as well as in the synovium of mice with OA and end-stage OA patients. In addition, vascular endothelial growth factor (VEGF) is an effective stimulator of angiogenesis and may also promote synovial fibrosis through extravasation [5]. These three are confirmed to be fibrogenic factors [6].

KOA is a complex disease, the pathogenesis of which involves not only mechanical but inflammatory and metabolic factors, leading to the destruction and failure of knee joints [7]. Each common osteoarthritis risk factor can lead to different mechanisms of KOA, such as increase of inflammatory factors [8] and oxidative stress. In addition, the extended metabolic characteristics of knee synovium contribute to imbalance of oxygen homeostasis and enhance hypoxia in the microenvironment [9]. Hypoxia-induced factor (HIF)-1α is recognized as a major regulator of hypoxia signaling, which mediates the adaptive response of cells to hypoxia by activating the transcription of genes encoding proteins. HIF-1α expression can be triggered in an inflammatory microenvironment even under normoxic conditions [10]. Clinical studies have shown that the HIF-1α concentrations in serum, synovial fluid and articular cartilage of patients with KOA are associated with progressive joint damage. It can be used as a biomarker for KOA progression and prognosis. HIF-1α is associated with the upregulation of genes encoding pro-inflammatory cytokines and growth factors, thereby activating fibroblasts and mediating fibrosis. Besides, the NLR family pyrin domain-containing 3 (NLRP3) inflammasome can be activated by HIF-1α [11]. The NLRP3 inflammasome has been implicated in the pathogenesis of a number of arthritic disorders, producing proinflammatory cytokines and degradative enzymes, such as interleukin-1 beta (IL-1β) which drives cartilage degeneration and synovial inflammation [12]. Inhibiting NLRP3 inflammasome can alleviate many types of fibrosis, especially synovial fibrosis [13,14].

Imperatorin (IMP) is a secondary metabolite of plants. It is one of furanocoumarin derivatives and widely used in many traditional Chinese herbal drugs (e.g. *Angelica dahurica*) which are employed to treat KOA. It has antitumor [15], antibacterial [16] and anti-inflammatory effects [17], but little is known about its role in the suppression of synovial inflammatory and fibrosis. Also, IMP is one of the active ingredients in ‘Yiceng’, a tropical drug used to treat KOA [18].

Therefore, in this study, we intended to assess the effects of IMP on synovial fibrosis induced by monosodium iodoacetate (MIA) *in vivo*, and an inflammatory model in primary synovial fibroblasts induced by lipopolysaccharide (LPS) *in vitro*. The therapeutic effect of IMP on synovitis and synovial fibrosis reveals it as a potential candidate for drug development.

## Materials and methods

### Reagents and antibodies

IMP (purity >99%) was purchased from Yuanye (Shanghai, China). MIA and dimethyl sulfoxide were obtained from Sigma (St Louis, USA). Antibodies against NLRP3, ASC, Caspase-1, VEGF, TIMP1, TGF-β1 and HIF-1α were purchased from Abcam (Cambridge, UK). HRP-conjugated affinipure goat anti-rabbit IgG(H + L) (Proteintech Group, Inc., SA00001-2, 1:20,000), HRP-conjugated affinipure goat anti-mouse IgG(H + L) (Proteintech Group, Inc., SA00001-1, 1:20,000). ECL luminescent liquid (Shanghai Tianneng, 180–5001), protein marker (Shanghai Tianneng, 180–6003), BCA protein assay kit (Thermo Fisher, 23,227). Bovine serum albumin, fetal bovine serum (FBS), Dulbecco’s Modified Eagle’s Medium (DMEM), TRIzol and 0.25% trypsin-EDTA were purchased from Gibco (Life Technologies Corp., California, USA). TransStart Green qPCR SuperMix was obtained from Takara (Dalian, China). The primers and rat GAPDH Endogenous Reference were supplied by Sangon Biotech (Shanghai, China). Enzyme-linked immunosorbent assays (ELISA) kits for IL-1β and IL-18 were supplied by Invitrogen (Life Technologies Corp., California, USA). All other chemicals were of reagent grade.

### Animals

Thirty 3-month-old SD male rats weighing from 250 to 290 g, 10 for each group (provided by Beijing Vital River Laboratory Animal Technology Co., Ltd.), were used for experimental KOA studies. The animals were maintained in a specific pathogen-free laminar-flow housing apparatus with controlled temperature, humidity, and 12 h light/dark regimen. All animal protocols were approved by the Animal Care and Use Committee of the Nanjing University of Chinese Medicine. All experiments were conducted in accordance with the National Institutes of Health Guidelines for the Care and Use of Laboratory Animals.

### Induction of KOA and drug administration

The rat osteoarthritis model was established according to a previous literature [6]. Thirty rats were randomly divided into three groups (Normal group, MIA group, MIA+IMP group). For the Normal group, injection of 50 µl 0.9% saline into articular joint was performed. For MIA group, 2 mg MIA dissolved in 50 µl 0.9% saline [11]. For MIA +IMP group, 5 mg/kg/day was selected as oral administration concentration from 2 weeks after injection as previously described [19]. IMP was dissolved in 0.5% carboxymethylcellulose sodium (CMC-Na), and 0.5% CMC-Na was given by intragastric administration alone for sham group and MIA group every day for 6 weeks until the rats were sacrificed. All rats were sacrificed 8 weeks after injection, and the synovial tissues were treated for histological analysis and further experiments.

### Histopathological analysis

For hematoxylin and eosin (H&E) staining, synovial tissues were frozen and fixed in 4% paraformaldehyde, soaked in 0.5 M EDTA and embedded in paraffin. The paraffin blocks were sectioned at a thickness of 5 μm [20].

### Isolation and primary culture of synovial fibroblasts

Primary rat synovial fibroblasts were obtained from additional normal rats. In brief, synovial tissues were washed 2–3 times with phosphate-buffered saline, minced into pieces of 2–3 mm^2^ and digested in 0.1% collagenase type II (Sigma Aldrich, St. Louis, MO, USA) for 30 min. Following cell dissociation, the samples were filtered through a cell strainer. After dissociation, fibroblasts were pelleted by centrifugation at 1500 rpm for 4 min and cultured in DMEM supplemented with 10% FBS (Gibco, Thermo Fisher Scientific, Waltham, MA, USA) and antibiotics (100 U/ml penicillin, 100 μg/ml streptomycin). The cells were cultured at 37°C in a humidified 95% air and 5% CO_2_ atmosphere. Passages 3–6 of the synovial fibroblasts were used for experiments.

Fibroblasts were treated with LPS (5 μg/ml) in DMEM for 6 h to stimulate inflammatory response and activate the NLRP3 inflammasome. Fibroblasts exposed to DMEM with the same volume of saline served as control. Before administration of LPS, IMP (50 μM) was used for 24 h or 48 h [21].

### Synovial extraction and preservation in rats

After 14 days of modeling or 14 days of treatment, the rats were sacrificed by CO_2_ asphyxia, and the knee joint hair was removed. The ligament was incised on both sides of the patellofemoral ligament. The upper edge of the humerus was transversely cut to the distal end of the quadriceps muscle until the femur. The free tibia and its surrounding tissue were opened until the distal end. The pale yellow translucent synovial membrane was discerned. The synovial tissue was carefully cut with a surgical blade. Paraformaldehyde was used for pathological sectioning, and the rest was placed in a cryotube at −70°C [22].

### Real-time PCR

RNA was isolated from synovial tissues and fibroblasts with Trizol (Invitrogen, CA, USA), respectively. Reverse transcription was performed by using a first-strand cDNA synthesis kit (Takara, Otsu, Japan) according to manufacturer’s instructions. qPCR was performed using Premix Ex Taq SYBR-Green PCR (Takara) according to manufacturer’s instructions on an ABI PRISM 7300 system (Applied Biosystems, Foster City, CA, USA).

Primers were designed and synthesized by Shanghai Biotechnology Service Co. Ltd. in accordance with the gene sequence in GenBank gene sequence design, together with Oligo v6.6. Sequences for primers are shown in Table 1. The mRNA levels of individual genes were normalized to that of GAPDH and calculated by the 2^−ΔΔCt^ method [23].

**Table 1.** Primer sequences

mRNA	Forward	Reverse
HIF-1α	5ʹ-CCGCAACTGCCACCACTGATG-3ʹ	5ʹ-TGAGGCTGTCCGACTGTGAGTAC-3’
NLRP3	5ʹ-GAGCTGGACCTCAGTGACAATGC-3’	5ʹ-ACCAATGCGAGATCCTGACAACAC-3’
ASC	5ʹ-AGAGTCTGGAGCTGTGGCTACTG-3’	5ʹ-ATGAGTGCTTGCCTGTGTTGGTC-3’
Caspase-1	5ʹ-ATGGCCGACAAGGTCCTGAGG-3’	5ʹ-GTGACATGATCGCACAGGTCTCG-3’
TGF-β1	5ʹ-GCAACAATTCCTGGCGTTACCTTG-3ʹ,	5ʹ-TGTATTCCGTCTCCTTGGTTCAGC-3’
TIMP1	5ʹ-GCGTTCTGCAACTCGGACCTG-3’	5ʹ-GTGTAGGCGAACCGGATATCTGTG-3’
VEGF	5ʹ-AGCGTTCACTGTGAGCCTTGTTC-3’	5ʹ-CCGCCTTGGCTTGTCACATCTG-3ʹ.

### Western blotting

The synovial tissue was dissected, weighed and mixed with RIPA lysate. The samples were centrifuged at 15,000 r/min for 15 minutes at 4°C. The cultured fibroblasts were washed and lysed. Protein concentrations were then quantified using the BCA protein assay kit. Protein samples were electrophoresed by SDS-PAGE to separate protein bands. The products were transferred from the gel to a PVDF membrane which was then blocked with 5% skimmed milk for 2 h. The membrane was incubated with polyclonal rabbit antibodies specific for NLRP3, caspase-1, ASC, TGF-β1, VEGF and TIMP-1 overnight at 4°C. On the next day, the membrane was incubated with secondary antibody for 2 h. The bands were visualized using the ECL method, and ImageJ software was used to quantify the total gray value (average gray value × gray value area) of protein band to calculate the relative value of target protein [24].

### ELISA

The peripheral serum of rats and culture supernatant of cells were collected and centrifuged at 10,000 rpm for 20 min at 4°C, after which the concentrations of IL-1β and IL-18 were measured by ELISA kits. All steps were performed according to the manufacturer’s instructions [25].

### Statistical analysis

Graphpad prism 8.0 software (San Diego, CA, USA) was used to statistically analyze the data. The measurement data were based on (χ ± s), and the numerical data were subjected to the χ^2^ test. The three groups of data conformed to normal distribution, and one-way ANOVA was used, including LSD and SNK tests. The Kruskal–Wallis *H* test was used for non-normal distribution. The difference was statistically significant when *P* < 0.05.

## Results

### Synovial fibrosis and hypoxia were found in MIA-induced KOA model rats

The NLRP3 inflammasome has been implicated in the pathogenesis of several arthritic disorders, producing degradative enzymes and proinflammatory cytokines to drive cartilage degeneration and synovial inflammation. Inhibiting NLRP3 inflammasome can mitigate many types of fibrosis, especially synovial fibrosis. Given that IMP has long been used to treat KOA, its effects on NLRP3 inflammasome cascade-induced synovial fibrosis and synovitis in KOA rats were assessed.

**Figure 1.**
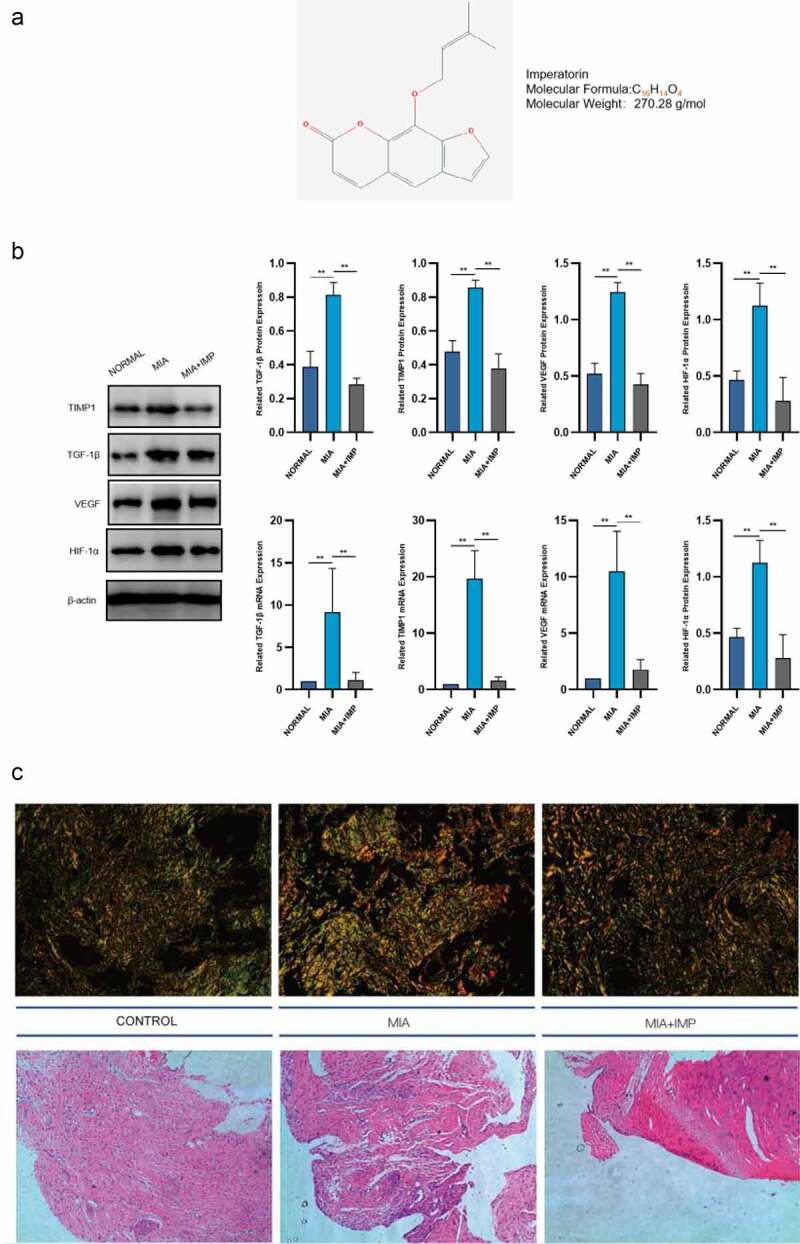
Fibrogenic and hypoxia markers were upregulated in MIA-induced KOA rats and downregulated by IMP. (a) Chemical structure of imperatorin. (b) mRNA and protein levels of HIF-1α, TGF-β1, TIMP1 and VEGF in Normal, MIA-induced KOA and KOA rats treated with IMP determined by qRT-PCR and Western blotting. (c) Representative synovium tissues undergoing Sirius red staining or H&E staining, 200×, scale bar = 100 μm. Values are represented as mean ± SEM (*p < 0.05, **p < 0.01)

The chemical structure of IMP is shown in Figure 1a. The upregulation of HIF-1α in pathological conditions suggested that the synovial membrane was under hypoxia conditions with synovial fibrosis induced by MIA in rats. HIF-1α protein and mRNA levels were markedly elevated in KOA synovial membranes (Figure 1b). The mRNA and protein expression levels of TGF-β1, TIMP1 and VEGF in fibrotic synovium were all upregulated in pathological conditions induced by MIA in rats (Figure 1b). The representative synovium tissues after staining are exhibited in Figure 1c.

### Activation of NLRP3 inflammasome in synovial membranes resulted in synovitis in rats

**Figure 2.**
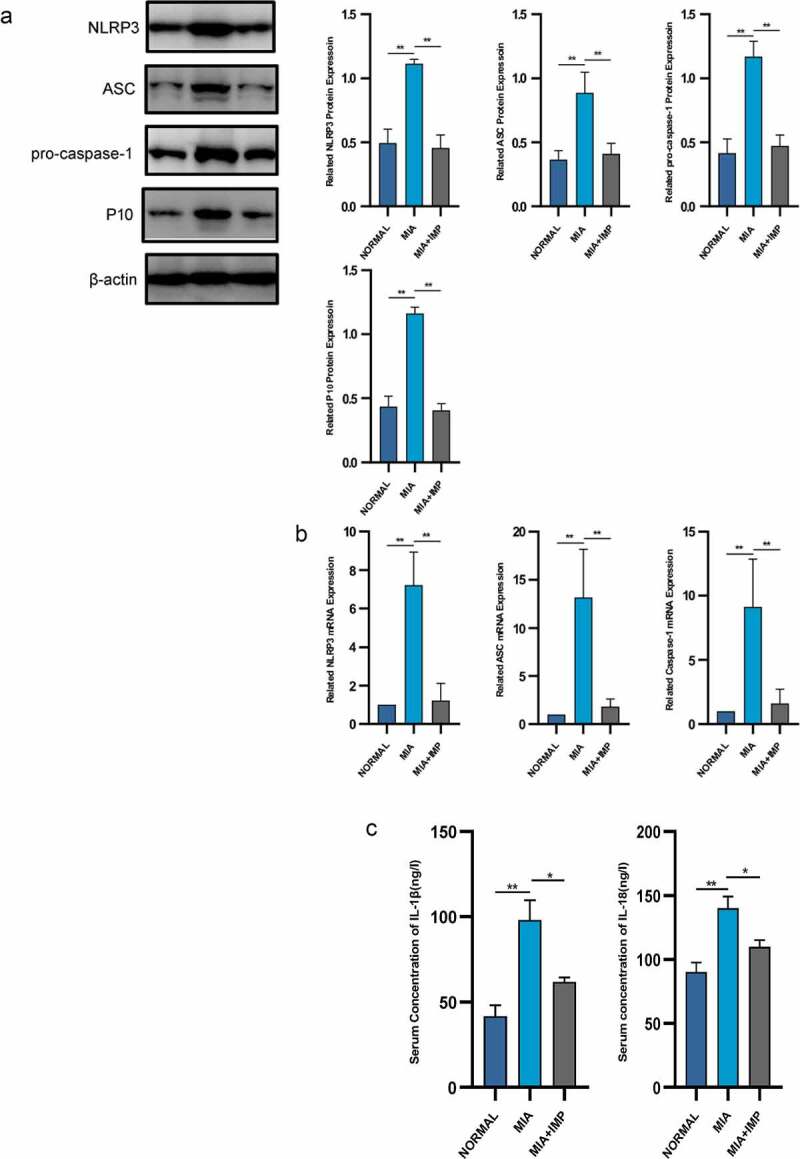
NLRP3 inflammasome was activated in MIA-induced KOA rats and partly inhibited by IMP. (a) Expression of NLRP3, ASC, pro-caspase-1 and P10 determined by Western blotting. The upregulation of NLRP3 inflammasome protein expression was inhibited by IMP. (b) mRNA levels were quantified by qRT-PCR in fibroblasts treated with or without LPS (5 μg/mL) or treated with LPS (5 μg/mL) and IMP. The upregulation of NLRP3 inflammasome mRNA expression was inhibited by IMP. (c) Serum concentrations of IL-1β and IL-18. Values are represented as mean ± SEM (*p < 0.05, **p < 0.01)

The key inflammatory factors of NLRP3 inflammasome-dependent cytokines, IL-1β and IL-18, were significantly upregulated in the MIA group compared with those of the NORMAL group (Figure 2c). Caspase-1 recruited by NLRP3 inflammasome cleaved pro-IL-1β and pro-IL-18 that promoted the maturation of inflammatory cytokines. We then evaluated the mRNA and protein expressions of NLRP3 inflammasome in the MIA group. Both mRNA and protein levels were upregulated compared to those of the NORMAL group (Figure 2a and b).

### IMP inhibited experimental synovitis and synovial fibrosis in rats

IMP, one of the representative components in furanocoumarin, is extracted from *Angelica dahurica*. The MIA+IMP group had milder resident cell hyperplasia, formation of lining cell layers and inflammatory infiltration than those of the MIA group. Sirius red staining showed that the collagen fibers were red, the nucleus was green, and other components were yellow. The MIA group showed more collagen fibers than those of the Control group, suggesting that IMP ameliorated synovitis and synovial fibrosis (Figure 1d). After IMP administration, the expressions of NLRP3, ASC, caspase-1 and caspase-1 p10 were determined by qRT-PCR and Western blotting. All these components related to NLRP3 inflammasome were significantly downregulated in MIA+IMP group compared with those of MIA group at both mRNA and protein levels. The production of caspase-1 (p10) was also significantly reduced by IMP compared with that of MIA group, as well as IL-1β and IL-18 (Figure 2a‒c). Besides, fibrogenic factors TGF-β1, TIMP-1 and VEGF were downregulated after IMP administration at both mRNA and protein levels (Figure 1c). Collectively, the administration of IMP inhibited synovitis, synovial fibrosis and hypoxia pathogenesis in rats.

### IMP inhibited LPS-induced upregulation of fibrogenic factors in fibroblasts

**Figure 3.**
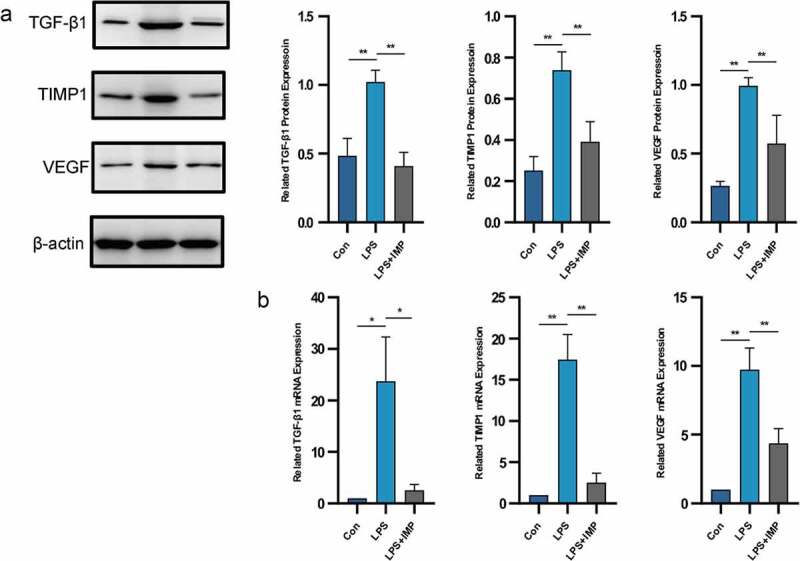
Fibrogenic marker expressions induced by LPS in fibroblasts were upregulated and downregulated by IMP. (a) Expression of TGF-β1, TIMP1 and VEGF determined by Western blotting. The protein expressions of these fibrogenic markers were inhibited by IMP. (b) mRNA levels were quantified by qRT-PCR in fibroblasts treated with or without LPS (5 μg/mL) or treated with LPS (5 μg/mL) and IMP (50 μM). The mRNA expressions of these fibrogenic markers were inhibited by IMP. Values are represented as mean ± SEM (*p < 0.05, **p < 0.01)

We next examined the *in vitro* role of IMP in LPS-stimulated primary cultured fibroblast-like synoviocytes. The upregulated expression of fibrogenic factors suggested their possible involvement in synovial fibrosis. Treatment of fibroblast-like synoviocytes with LPS (5 μg/mL) significantly upregulated TGF-β1, TIMP-1 and VEGF at both mRNA and protein levels. These fibrogenic factors contributed to synovial fibrosis *in vivo*. After IMP administration, all those factors were downregulated significantly (Figure 3a and b).

### IMP inhibited activation of NLRP3 inflammasome in LPS-stimulated fibroblasts

**Figure 4.**
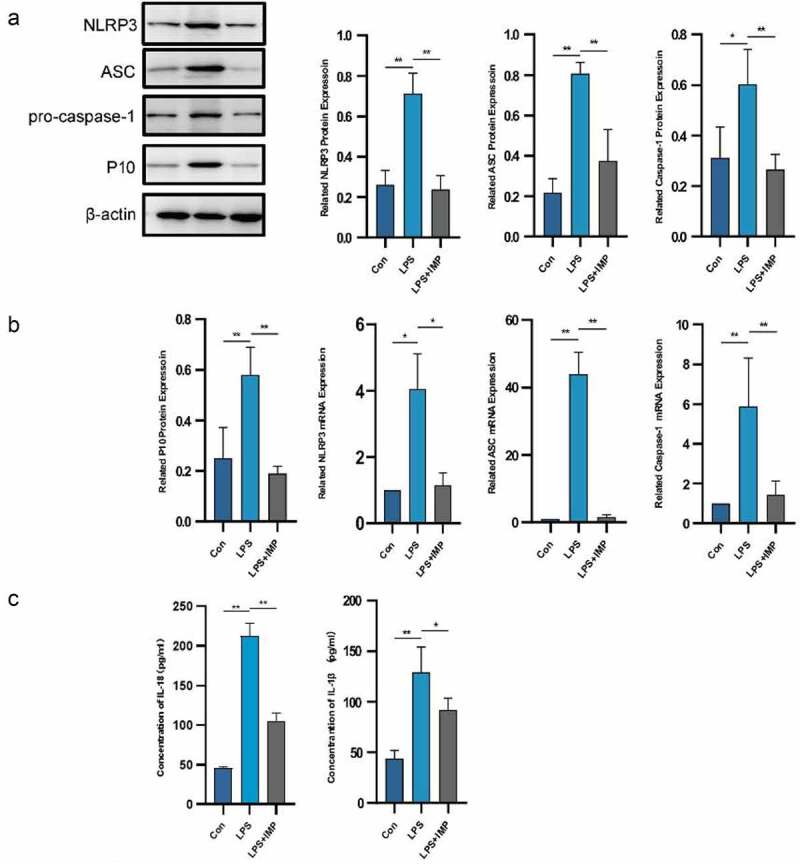
NLRP3 inflammasome was activated by LPS in fibroblasts and partly inhibited by IMP. (a) Expression of NLRP3, ASC, pro-caspase-1 and P10 determined by Western blotting. The protein expression levels were inhibited by IMP compared to those in the LPS group. (b) The mRNA levels were quantified by qRT-PCR in fibroblasts treated with or without LPS (5 μg/mL) or treated with LPS (5 μg/mL) and IMP (50 μM). The mRNA expression levels were inhibited by IMP compared to those in LPS group. Values are represented as mean ± SEM (*p < 0.05, **p < 0.01). (c) Inflammation cytokines IL-1β and IL-18 were measured by ELISA. Values are represented as mean ± SEM (*p < 0.05, **p < 0.01)

NLRP3, ASC and caspase-1 expressions were significantly upregulated in the LPS group compared with those of the NORMAL group at both mRNA and protein levels. IMP significantly inhibited the mRNA and protein expressions of NLRP3 inflammasome compared with those of the LPS group. The production of cleaved caspase-1 and caspase-1 p10 was also confirmed by Western blotting and qRT-PCR. It showed significant upregulation in the LPS group compared with that of the NORMAL group, which was suppressed by IMP (Figure 4a and b). The downstream production of IL-1α and IL-18 was upregulated after LPS stimulation, and the inflammatory cytokines secreted by fibroblasts were downregulated by IMP (Figure 4c).

## Discussion

Hypoxia, inflammation and fibrosis persistently exist in the pathological progress of KOA [26]. Herein, we demonstrated the therapeutic effects of IMP on KOA. IMP significantly inhibited the expressions of HIF-1α mRNA and protein, activated the NLRP3 inflammasome, and downregulated the levels of fibrogenic factors in KOA. KOA is characterized by the progressive destruction of articular cartilage and surrounding tissues, especially synovial tissue [27]. In this study, we verified for the first time that IMP intervened with the pathological processes of KOA. The therapeutic effects of IMP may be related to the suppression of inflammation and synovial fibrosis.

Increased HIF-1α is widely involved in the progression of diseases, as well as synovial fibrosis and inflammation. It is well established that low oxygen tension exists in the synovium when KOA occurs because of synovial angiogenesis and inflammatory cell infiltration [28]. Hypoxia in the microenvironment is mainly marked by HIF-1α expression. Compared with normal rats, the expressions of HIF-1α and its target genes VEGF and TIMP-1 in synovial tissue significantly increased in KOA group [29]. We inferred that the inhibition of HIF-1α expression suppressed synovial fibrosis. In addition, HIF-1α can regulate NLRP3 expression. In the case of hypoxia, the expressions of HIF-1α, NLRP3, caspase-1 and IL-1β increased [30]. HIF-1α is known to regulate a plethora of human diseases. By regulating NLRP3 (transcript) expression under these conditions, it becomes a key node in linking hypoxia response to pro-inflammatory status. Increased expression of NLRP3 and enzymatic activation of caspase-1 are one of the conditions for the upregulation of IL-1β and IL-18. These increased inflammatory factors were driven by the NLRP3 inflammasome. We found that IMP inhibited not only the expression of HIF-1α in fibroblasts, but also the activation of NLRP3 inflammasome and the expressions of downstream IL-1β and IL-18. Inhibiting HIF-1α can lead to synovial fibrosis in KOA rats; so, we observed the changes of three fibrogenic factors (TGF-β1, VEGF and TIMP-1) at the same time.

Synovial fibrosis is a pathological process observed in several musculoskeletal diseases. HIF-1α regulates the expressions of genes and proteins related to angiogenic growth factors, such as VEGF and TGF-β1 [31]. TGF-β1 is one of the major indicators of synovial fibrosis that activate myoblasts, promote ECM gene expression, and inhibit ECM degradation [32]. However, the triggering mechanism of synovial fibrosis in the knee joint remains largely unknown. Ko et al. found that TIMP1 was upregulated in OA fibroblasts stimulated with TGF-β1 and in mice with TGF-β-induced fibrosis [33]. TIMP-1 is an inhibitor of matrix metalloproteinases, which is also elevated in the synovium of end-stage OA patients [5]. TIMP-1 is induced by TGF-β, usually as an accelerator of fibrosis development [34]. A recent study showed that the articular capsule of fixed knee joint was in the hypoxia state, and VEGF was upregulated at the mRNA and protein levels after immobilization. The decoy ODN transfected with HIF-1 successfully suppressed the transcriptional activation of HIF-1. VEGF expression was subsequently suppressed [35]. Upon RA, hypoxia is caused by increasing metabolic requirements for white blood cells to enter RA joints, which can cause HIF-1α to accumulate in the cytoplasm and induce VEGF expression. Positive feedback regulation of the HIF-1α and VEGF pathways can trigger angiogenesis during hypoxia. In addition, the levels of VEGF and HIF-1α in synovial tissue were positively correlated with microvascular density [36]. In this study, IMP decreased TGF-β1 and TIMP1 mRNA and protein levels *in vitro* and *in vivo*. Therefore, IMP may inhibit synovial fibrosis by suppressing the expressions of fibrogenic factors.

IMP is one of the furanocoumarin derivatives and exists in many Chinese herbal drugs with anti-tumor, antibacterial, cardiovascular, anti-inflammatory activities. In our previous studies, we found that synovial fibrosis was highly correlated with the activation of HIF-1α and NLRP3 inflammasome. These results indicate that IMP can mitigate synovitis and synovial fibrosis by inhibiting HIF-1α/NLRP3 inflammasome signaling.

## Conclusions

In summary, IMP can alleviate synovial hypoxia and synovitis, and thus MIA-induced synovial fibrosis in KOA rats. It can reduce the release of inflammatory mediators and upregulate fibrosis markers induced by MIA or LPS by inhibiting the activation of NLRP3 inflammasome. Taken together, IMP may be a potentially effective therapy for KOA, especially for synovial fibrosis. Further studies are required to determine the signal pathway involved in HIF-1α/NLRP3 inflammasome activation/fibrosis. Its mechanism provides new ideas for treating KOA-related synovitis and synovial fibrosis.

## Data Availability

The datasets used and/or analyzed during the current study are available from the corresponding author on reasonable request.

## Abbreviations

CMC-Na: carboxymethylcellulose sodium; ECM: extracellular matrix; FBD: fetal bovine serum; H&E: hematoxylin and eosin; HIF: hypoxia-induced factor; IL: interleukin; IMP: imperatorin; KOA: knee osteoarthritis; LPS: lipopolysaccharide; MIA: monosodium iodoacetate; TIMP: tissue matrix metalloproteinase; VEGF: vascular endothelial growth factor.

## Highlights

1. KOA synovium shows both inflammatory and fibrotic phenotypes.

2. Imperatorin relieves inflammatory and fibrotic phenotypes in LPS-induced FLSs.

3. Imperatorin alleviates synovitis and synovial fibrosis in MIA-induced KOA rats.
